# Aorto-aortic bypass in an infant with middle aortic syndrome and Marfan syndrome: a 15-year follow-up

**DOI:** 10.1093/icvts/ivad011

**Published:** 2023-01-19

**Authors:** Roland Heck, Björn Fischer-Zirnsak, Joachim Photiadis, Denise Horn, Petra Gehle

**Affiliations:** Department of Cardiothoracic and Vascular Surgery, German Heart Center Berlin, Berlin, Germany; Department of Medical Genetics and Human Genetics, Charité – Universitätsmedizin Berlin, corporate member of Freie Universität Berlin, Humboldt Universität zu Berlin and Berlin Institute of Health, Berlin, Germany; Max Planck Institute for Molecular Genetics FG Development and Disease, Berlin, Germany; Department of Congenital Heart Surgery—Pediatric Heart Surgery, German Heart Center Berlin, Berlin, Germany; Department of Medical Genetics and Human Genetics, Charité – Universitätsmedizin Berlin, corporate member of Freie Universität Berlin, Humboldt Universität zu Berlin and Berlin Institute of Health, Berlin, Germany; Department of Cardiology, Charité – Universitätsmedizin Berlin, Berlin, Germany

**Keywords:** Great vessel anomaly, Aortic Surgery, Marfan Syndrome, Genetics, Hormone Therapy

## Abstract

We present a 15-year follow-up after aorto-aortic bypass surgery in a 7-month-old infant with middle aortic syndrome and confirmed Marfan syndrome. In anticipation of her growth, the length of the graft was adjusted to the anticipated length of the narrowed aorta in her adolescence. In addition, her height was controlled by oestrogen, and her growth was stopped at 178 cm. To date, the patient is free from aortic reoperation and lower limb malperfusion.

## INTRODUCTION

Since Marfan syndrome (MS) was first described clinically, its genotype and epigenetic consequences for the aorta and the myocardium have been increasingly investigated over the past decade [1]. The clinical courses of affected patients were described in larger cohorts when planning therapy to prevent adverse outcomes. The middle aortic syndrome (MAS) is characterized primarily by segmental narrowing of the descending aorta. Its aetiology ranges from idiopathic narrowing due to a failed fusion of the 2 dorsal aortas to secondarily achieved aortic narrowing due to Takayasús arteritis or neurofibromatosis type 1 [2, 3]. To the best of our knowledge, a combination of those 2 rare diseases has not been documented. The goal of this article was to present this severe case of MAS in a patient with genetically confirmed MS.

## CASE REPORT

A 7-month-old female patient (size: 73 cm; weight: 7.05 kg) was transferred to our centre with suspected post-ductal, non-cyanotic aortic isthmus stenosis. Consent for publication was granted by the patient and her parents. She presented with a pulse deficit of the lower extremities, upper body hypertension stage II (risk ratio 140/84 mmHg; ≥ 5 mmHg above the 99^th^ percentile for her age), lower body relative hypotension (risk ratio 85/72 mmHg), a dilated left ventricle (28 mm) with impaired left ventricular function and mitral prolapse with consecutive grade 2–3 mitral regurgitation [4].

**Figure 1:**
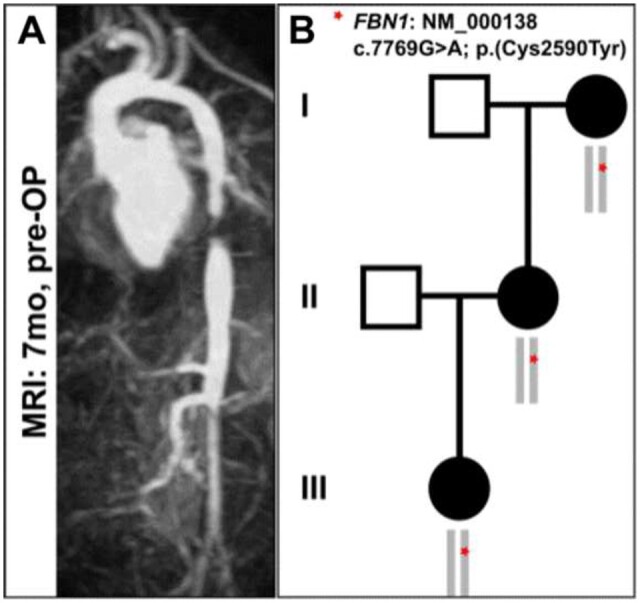
(**A**) Preoperative magnetic resonance image. (**B**) Family tree of the patient in respect to the FBN1 mutation.

Via magnetic resonance imaging and angiography, a 2-cm long and 1.7-mm wide stenosis of the descending aorta was diagnosed (Fig. 1A). The initial attempt of balloon dilatation of the stenotic segment failed due to rigid wall conditions and had no positive functional effect. Genetic diagnostics were performed due to positive family anamnesis (Fig. 1B).The operation was performed via a left thoracotomy in the fourth intercostal space. The proximal descending aorta was clamped tangentially and a 10-mm Gore-Tex graft was anastomosed end to side to the proximal and distal normally dimensioned segments of the aorta. The liberally sized length of the graft was “folded” into the thoracic cavity to be able to “unfold” during the longitudinal growth of the patient. After declamping, there was no blood pressure difference between the upper and lower body.

**Figure 2:**
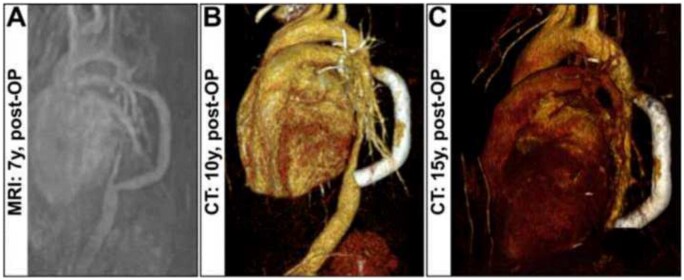
(**A**) Magnetic resonance image obtained at 7 years of age. (**B**) Computed tomography at 10 years of age. (**C**) Computed tomography at 15 years of age. CT: computed tomography; MRI: magnetic resonance imaging; post-OP: postoperative; y: year.

Postoperative ultrasound showed laminar flow in the graft and peri-anastomotic segments (Fig. 2A). Angiography showed a well-chosen graft size and competent and open anastomoses (Fig. 2B). The biventricular function regenerated as did the LV dilatation; the MI decreased to grade 1 with persisting prolapse.

During the period between the operation during infancy, when the patient had a body size of 73 cm and her adolescence, when she had a total body size of 178 cm, the aortic dimensions changed dramatically (Fig. 2 A-C). To date, the patient does not show any signs of claudication, and no pathological pressure gradient was measured between the upper and the lower extremities. Early operative intervention prevented secondary organ failure and secondary arterial hypertension. The narrowed aortic segment has shown steady growth in length and width. In the current computed tomography scan, the diameter of the native aortic segment increased from 1.7 mm to 8 mm. At the skeletal age of 13.5 years and an expected ongoing longitudinal growth of 6 cm, the indication for growth-inhibiting therapy was set, because growth at the end of adolescence occurs predominantly in the torso, which was highly relevant for the stretched stent graft. Therapy was initiated at the age of 14 years and 2 months with an ongoing daily dose of 100 µg ethinyl estradiol. Every 4 weeks we added a daily dose of 10 mg of medroxyprogesterone acetate for 7 days [5]. We continued with the described protocol until the termination of growth at the skeletal age of 16 to 17 years.

In 3 generations, the pathogenic *FBN1*: NM_000138 variant c.7769G>A identified by Sanger sequencing segregates with the disease. This variant is predicted to cause a missense alteration affecting residue 2590 of FBN1 (p. Cys2590Tyr). This variant has been described previously in the literature as causative for Marfan syndrome, and it is annotated in the Human Gene Mutation Database under the accession number CM098728 [6].

## COMMENT

To the best of our knowledge, MAS is not related to MS; however, genetic disorders like neurofibromatosis 1 and Williams syndrome are associated with MAS, especially when the stenosed segment is located suprarenally [3]. In our patient, both the laboratory values and the clinical presentation showed no evidence of inflammatory vascular disease such as Takayasu's arteritis, which would have required preparatory conservative drug therapy prior to surgery [7]. Although the mean detection age is 7.1 years and various interventional and surgical options can be discussed, the severity of MAS increases the younger the patients are when MAS is diagnosed [2]. Medical therapy is successful only in patients with mild to moderate MAS, whereas in severe MAS, medical therapy requires invasive treatment [8]. Endovascular approaches are an accepted option, especially for acute relief of resistance, but they have a failure rate and indication for reintervention in 28%. Surgical therapies including patch aortoplasty and aorto-aortic graft implants have a mortality rate of up to 4%, but the reported freedom from reintervention is 72% at 10 years follow-up [3]. In endovascular intervention, longitudinal growth increases the risk of intimal tears and dissection [8].

In our case, postponing the intervention was not an option due to left ventricular dilatation and already impaired left ventricular function [9]. The main challenge was the suspected extensive longitudinal growth (motheŕs size: 191 cm). Due to the expected growth, the length of the graft was longer than needed at the time of surgery to enable an elongation during longitudinal growth. A patch reconstruction was not considered because of a lack of native aortic tissue in the affected segment. A reoperation would have been indicated in advance. Mobilization and approximation of the 2 aortic segments would have been the strategy of choice in a 7-month-old infant, especially to avoid artificial graft material. After possible approximation, increased tension of the anastomosis was expected. In an infant with a diagnosed FBN-1 mutation, this fact was crucial in the decision to perform an aorto-aortic bypass. The ability to use tangential clamping, thereby avoiding use of the heart-lung machine for the 2 end-to-side anastomoses, also favoured the bypass.

The stenosed aortic segment has increased significantly in diameter and length to this day. On the one hand, these increases could be an expression of the residual growth capacity of the stenosed portion of the tissue. Further calcification with concomitant narrowing of the graft and the anastomosis would make reoperation necessary. As long as clinical signs of lower limb malperfusion and upper and lower limb blood pressure differences are absent, we will not consider further invasive diagnostics and/or surgical therapy in terms of a thoraco-abdominal replacement. Follow-up imaging should take place every 3 years or earlier if there is evidence of clinical impairment. On the other hand, having the diagnosis of Marfan syndrome, we may have to interpret the increase in diameter of more than 450% as a developing aortic aneurysm. In this case, replacement of the descending aorta in adulthood should be considered now.

## DISCLOSURES

None of the authors has anything to disclose.
